# What older adults find meaningful in organised sport participation: proposing the HeRO model

**DOI:** 10.3389/fspor.2026.1760024

**Published:** 2026-06-22

**Authors:** Susanna Geidne, Helena Ericson, Mikael Quennerstedt

**Affiliations:** 1 Faculty of Medicine and Health, School of Health Sciences, Örebro University, Örebro, Sweden; 2 The Swedish School of Sport and Health Sciences (GIH), Stockholm, Sweden

**Keywords:** health promotion, health resources, salutogenic theory, settings-based, sports clubs

## Abstract

**Introduction:**

Few older adults participate in organised sport. The reasons highlighted have predominantly focused on physical health factors related to the effects of physical activity. We argue that what, who, and how we ask matters in understanding more about older adults’ participation in sport. We need more knowledge regarding how participation relates to health beyond purely the physiological effects of physical activity. The aim of this study was therefore to contribute knowledge about which health resources older adults find meaningful for participation in organised sport.

**Method:**

This study applied salutogenic theory to identify health resources through a cross-sectional survey. The sample included more than 1,000 sports clubs in Sweden (*n* = 4,837 older adults, 60−96 years of age, one-third women, participating in 50 different sports). Open-ended responses from the Salutogenic Physical Activity Health Resources Questionnaire were analysed using a theory-driven approach.

**Results and conclusion:**

We propose HeRO, an empirically based conceptual model, to explain older adults’ participation in organised sport. Participation can be explored and understood as a combination of health resources that people consider meaningful. Ten health resources were identified, with the majority of respondents highlighting having fun, socialising with others, maintaining sport as a routine in their lives, and feeling fit as central to their participation.

## 1 Introduction

Physical activity is one of the most beneficial strategies for people of all ages to maintain overall health, mobility, and independence (1). Organised sport provides a setting in which older adults can be physically active while also engaging in social activities. However, relatively few older adults participate in sport, and participation tends to decline with age (2, 3), raising concerns for public health and primary care for older adults.

As reported in other studies, reasons highlighted by older adults who participate in sport are predominantly related to physical health factors stemming from physical activity (3–6), along with social factors such as social contact, fostering relationships, and promoting regular engagement (3, 5–7). Less frequently reported are age-related factors, such as negotiating the ageing process and coping with ageing (4, 6, 7), and general participation factors such as capability, motivation, meaningfulness, and enjoyment (8, 9). A few studies also emphasised factors related to sport that go beyond physical activity, including the value of competition (5, 10), volunteering through sport (4), and intergenerational bonding for families (3).

While many of these studies provide crucial and wide-ranging evidence regarding participation in physical activity among older adults, more knowledge is needed on how participation in sport is related to health beyond the effects of physical activity, and specifically which health resources older adults find meaningful for participation in organised sport (11–13). We also need robust, theoretically grounded methodologies for exploring these issues. In this study, we apply a salutogenic perspective and the concept of health resources (11) to explore what older adults find meaningful about participation in organised sport in order to better build cumulative knowledge in the field. Information on this topic has been lacking due to three distinct issues that potentially influenced the results of previous studies: (i) what counts as organised sport, (ii) who is defined as an older adult, and (iii) what questions the studies asked.

First, the definition of sport varies across studies. Some studies focus on physical activity, some on organised physical activity, and some on organised sport. The same type of activity can thus be included in all these studies (e.g., running), but they can also differ (e.g., walking alone compared to organised walking football). What is included in sport can also vary across countries. For example, Sweden has a broad definition of what is considered a sport (including both non-competitive and competitive activities), especially for target groups such as children and older adults (14). This is important for sampling older adults in studies and for identifying reasons for participation beyond physical activity.

Second, the reasons people participate in sport also depend on who is asked. Older adults represent a large target group spanning an age range of up to 40 years, with varied backgrounds, physical status, gender, and prior engagement in physical activity, among other factors. Some studies have examined the experiences of older adults who have never participated in sport (2, 15), while others have focused on older adults who participate in individual competitive sport, such as Masters games (5). Hence, although this research is vital for understanding inactivity, non-participation, and participation in highly competitive environments, less is known about why older adults engage in and continue participating in organised sport as a heterogeneous group.

Third, the reasons provided by older adults regarding their participation in sport have much to do with what researchers ask them, particularly in relation to health. In the studies reviewed for this article, we encountered various concepts, perspectives, theories, and methods. Studies from diverse traditions ask different questions influences by their disciplinary backgrounds, theoretical frameworks, and methodologies. One way of asking questions is about what influences participation. Here, the theoretical perspective, the COM-B model, hypothesises that participation is influenced by capability, opportunity, and motivation (8, 9). In their review, Stenner et al. (5) explored the reasons for participation and the motivators involved, drawing on studies with varied methodologies and data. Moreover, the concept of health is used differently across studies, with many studies on the relationship between health and sport in older adults being based on understanding what causes illness rather than what promotes health (16).

In sum, what we ask, who we ask, and how we ask matter if we want to understand older adults’ participation in organised sport, which is important if more older adults are to participate in sport. Many of the mentioned studies on older adults have also concluded that there is a need for further theory development taking into consideration age-related differences and perspectives of understudied populations, particularly in relation to health (17). Responding to these calls, this study investigates what older adults find meaningful through theoretically developed health resources—a perspective that focuses on what promotes health in a broad sense (16). Initially developed from a small group of older women in resistance training (11), then tested in a regional small-scale study of organised physical activity initiatives (18), these health resources are further developed in this study using a large sample of almost 5,000 older adults—both men and women across a large age span—participating in organised sport in Sweden. Hence, the aim of this study is to contribute knowledge about what health resources older adults find meaningful for participation in organised sport. The specific research questions are as follows:

- Which health resources in relation to participation in organised sport can be confirmed and identified in a Swedish sample of older adults?
- Which characteristics of each health resource can be identified?

We also propose an empirically based conceptual model—HeRO—as a tool for exploring and understanding different health resources, incorporating both individual and organisational aspects of participation.

## 2 Materials and methods

In this study, we adopt a health-promoting perspective, specifically salutogenic theory, to identify health resources (12). A central feature of salutogenic perspectives is that the relationship between health and non-health is not viewed as an either–or dichotomy—something one has or does not have—but rather as varying levels of health that are constantly created and maintained through a process. Therefore, exploring health involves asking why people are healthy or what can be done within specific institutions to further develop health. In the study, we use the concept of health resources, described by McCuaig and Quennerstedt (13) as “… different ways in which people from different backgrounds and in diverse contexts draw upon different resources to live a good life.” By focusing on health resources, we examine factors related to health in sport, considering both older adults’ participation in organised sport and the nature of the sporting activities in which they engage (11, 19). Although organisational features are important, this article emphasises the health resources that older adults find meaningful for participation.

Data in this study were generated through an evaluation assigned by the Swedish Sports Confederation (RF) (14). Data were collected from a sample comprising all sports clubs in Sweden that, during winter 2020/2021, applied for funding from RF for organised sport for older adults. The sample included more than 1,000 sports clubs, with a wide range in the number of older adults participating in each club. In February 2021, the sports clubs were contacted and asked to forward a survey to all their members aged 60 or older. This yielded responses from 4,837 older adults. Approximately one-third of respondents were women. The highest percentage was in the 65–70-year age group, with several respondents over 90 years of age. The majority of participants were born in Sweden (96%), and over 80% met the global physical activity recommendations. They represented nearly 560 sports clubs and participated in over 50 different sports.

The study utilised the Salutogenic Physical Activity Health Resources Questionnaire (SPAHRQ), focusing on variables related to health resources. The study adhered to the Swedish Research Council’s ethical principles of good research practice. Participants were informed in writing and provided written consent. No personal or sensitive data were collected. The variables were based on a theory-driven approach (13) to explore which health resources participants characterised as important for maintaining physical activity. These variables were previously identified in an in-depth study (11) and subsequently tested in a smaller-sample study (18). The seven specific health resources are (i) social relations, (ii) positive energy, (iii) self-worth, (iv) capability in and about physical activity, (v) the habit of exercising, (vi) identity as an exercising person, and (vii) embodied satisfaction. These seven previously identified health resources were operationalised as questions in the SPAHRQ, which asked older adults what aspects of their ongoing organised activities they found to be meaningful (see Table 1). Answer options were dichotomous, i.e., meaningful/not meaningful. Furthermore, respondents could select the box “Other” (approximately 5% answered this) and provide open-ended responses (389 chose to answer this non-mandatory option).

**Table 1:** An overview of the identified health resources and their characteristics (*n* = 4,837). The original health resources are based on the full sample. The new health resources are derived from 389 open-ended answers.

Health Resources	Characteristics	Agreement (in %)
***Confirmed health resources***		
HR1. Positive energy and enjoyment	Enjoyment and wellbeing here and now, as well as the energy the activities provide during the rest of the day.	84
HR2. Social relations	Relationships with friends during and after activities, caring for others, and giving back to the organisation.	81
HR3. The habit of exercising	Functionality in everyday life and routines when doing physical activities on a regular basis to prevent various ailments connected to ageing.	79
HR4. Embodied satisfaction	Feeling good about the body and an embodied satisfaction relating to physical fitness as well as physical appearance, and looking good.	76
HR5. Self-worth	The confidence and autonomy that participation in organised sport leads to.	32
HR6. Identity as an exercising person	Identity as someone doing physical activity regularly, as well as being a role model for other older adults to participate in organised sport.	21
HR7. Capability in and about physical activity	Learning and developing physical and mental skills, performing better, and taking on a challenge.	25
***New health resources (based on 389 open-ended answers)***		
HR8. Competing	Competing against oneself and others, and the suspense that competing offers.	Half of the comments
HR9. Nature experiences	Positive relationship with nature when participating in sport activities, and the value of being outdoors.	A fourth of the comments
HR10. Relationship with animals	Interplay with animals where the relationship with the animal is meaningful in itself when being physically active.	A few comments

### 2.1 Analysis

As a point of departure, we used previously identified health resources (11), which were explored through the following questions: How do the participants describe physical activity as meaningful in their daily lives? How do the participants describe physical activity as a resource for managing daily life? How do participants comprehend physical activity in their daily lives? In answering our research questions in the present study, we used three analytical questions to align with our purpose and to be able to structure our analytical procedures: (1) How can previously identified health resources be described and specified? (2) What other health resources can be identified? (3) In what ways do results from questions 1 and 2 confirm or modify previously identified health resources?

The first analytical question was used deductively, with open-ended comments linked to the original seven health resources in the survey. Each health resource was then inductively analysed to describe and specify dimensions within it. This process employed a previously developed deliberative strategy for salutogenic qualitative analysis (11). The aim of this strategy was to achieve collective agreement, where all co-authors have the opportunity to offer judgements on different ideas, views, and arguments. The strategy involves deliberation on quality end goals [i.e., worthy topic, rich rigour, sincerity, credibility, resonance, significant contribution, ethical, and meaningful coherence which are central to the research's rigour (20)]. The deliberation process ensured that, at each step, agreements about each dimension within the health resources were credible, resonated with previous studies (11), contributed to confirming and specifying each resource, and maintained coherence within and between the resources. The second analytical question focused on participants who selected the “Other” option and aimed to identify additional health resources, applying the same analytical strategy described earlier. In this phase, emphasis was placed on comments that did not align with the initially identified resources to discover what other aspects of participation in organised sport were regarded as meaningful. In this process, three distinct patterns were identified: competing, nature experiences, and relationship with animals. These were identified as separate new health resources since what some older adults found meaningful varied from previously identified resources. The third analytical question compared earlier health resources from Ericson et al. (2018) with the present larger dataset and sought to confirm similarities or describe modifications. These modifications were categorised as (i) confirmed and nuanced, (ii) confirmed and extended, or (iii) new health resource. The results of this third analysis are presented in the Discussion section.

## 3 Results

This study identified and confirmed 10 health resources that older adults found meaningful for participation in organised sport (Table 1). In the following, we describe these 10 health resources and their different characteristics, using illustrative examples from the data.

### 3.1 HR1—positive energy and enjoyment

The health resource “positive energy and enjoyment” was the most frequently reported resource among participants (84%). This health resource reflects participants’ immediate wellbeing, in the here and now, when the activity is taking place. It is also about forgetting daily worries, experiencing joy, and being in an improved mood for the rest of the day. Hence, this health resource fosters a positive feeling in participants during and after a training session, generating positive energy.

Wellbeing through participation in organised sport was also described as crucial for living a good life.

What else should you do to live a good life? (Woman, Orienteering, age 60–64)

I feel enjoyment, and I forget all the troubles of everyday life. (Man, Orienteering, age 65–70)

The focus of this health resource is enjoyment in the moment—the fun of doing activities—and how this enjoyment translates into positive energy felt both in the body and mind.

My whole person feels good. It is like going into a state without time and space and coming out as a new person. (Man, Budo, age 65–70)

I am feeling reborn after every training session. (Woman, Gymnastics, age 71–75)

The positive energy the activities generate through enjoyment also extends to cognitive wellbeing and better mood throughout the day and in life in general.

Clearing my mind, like emptying the hard drive, and sometimes getting a perspective on what you do and value in life. (Man, Equestrian sports, age 60–64)

Helps me to cope and still be happy after I became a widow. (Woman, Skiing, age 60–64)

In sum, positive energy and enjoyment is the most commonly identified health resource among older adults in organised sport. The health resource includes enjoyment and wellbeing here and now, as well as the energy the activities entail during the rest of the day.

### 3.2 HR2—social relations

The health resource “social relations” was the second most frequent resource among the participants in this study (81%). The resource is about experiencing a sense of community, meeting friends, and being active together. Examples include having coffee and chatting—called “fika” in Sweden—and taking a sauna together after an activity. These friendships may be old ones or new and can include intergenerational sport companions. Caring for others is an essential aspect of this health resource, involving both caring for friends and supporting close ones at home. Another social aspect is giving back to the sports club through coaching or volunteer work. The importance of meeting good leaders is also emphasised.

Socialising through organised activities involves both interactions during the activity and interactions before and after the activity.

Meeting friends in the club, feel how the muscles relax during movement in the warm water and the relaxation in the Sauna afterwards. (Woman, parasport, age 76–80)

Another important characteristic of Swedish organised sport for older adults is having a coffee together and chatting after the activity, called “fika.”

We always do ‘fika’ in the clubhouse after exercise. (Man, Tennis, age 65–70)

Social relations also involve caring for others. At this stage of life, caring for one another becomes very visible in everyday life, as close friends and partners often need care.

We care for each other. We are there in both joyful moments and in sorrow. (Woman, Golf, age 60–64)

Leaders and coaches, as well as coaching activities yourself, are also meaningful. This includes the social relations created as a result of coaching, which make participation meaningful for some.

I have been and am a leader, which feels good when other people enjoy being with us. Some new participants 85 + now can have a meaningful life. (Woman, Boule, age 81–84)

In sum, social relations is one of the most important health resources older adults find meaningful in organised sport. It involves maintaining relationships with friends during and after activities, caring for others, and giving back to the organisation.

### 3.3 HR3—the habit of exercising

The health resource “the habit of exercising” involves routines and the continuous regularity of doing exercise in everyday life. This was rated as meaningful by a large proportion of participants (79%). The regularity of doing exercise is about delaying the ageing process and the various benefits of regular physical activity, including improved functionality, reduced pain, better sleep, prevention of diseases, and rehabilitation after injuries and surgery. The habit of exercising is thus seen as an investment in physical health.

This health resource involves engaging in different physical activities regularly, which is helpful in maintaining physical health and in maintaining routines, particularly as a retired person.

As retired, it is also important to have daily routines and recurring activities in life. (Woman, soccer, age 65–70)

Regularly participating in exercise becomes a way to maintain your health and uphold bodily functionality to cope with everyday life.

I feel that I become stronger and that the greyness of everyday life has become easier. If I am fit, it is considerably easier to carry the grocery bags home, it is easier to chop wood for the fireplace, and gardening at the cottage becomes easier. (Man, orienteering, age 65–70)

Regular exercise leads to delayed signs of ageing, a fit body and mind, better functionality in terms of balance, muscular strength, general mobility, and cognitive functioning.

To slow down the ageing of body and mind. (Man, Track and field, age 65–70)

Not losing muscle mass. Counteract ageing through a cascade of hormonal betterments. (Man, Track and field, age 71–75)

Another critical aspect of the habit of exercising is preventing illness and injuries, reducing pain from previous injuries, and thus living a good life despite various struggles that come with ageing.

Helps me to avoid pain in my back, neck and shoulders. (Woman, Gymnastics, age 65–70)

In sum, the health resource the habit of exercising reflects functionality in everyday life and a routine that makes physical activities meaningful, thereby preventing various ailments associated with ageing.

### 3.4 HR4—embodied satisfaction

The health resource “embodied satisfaction” was rated as meaningful by a large proportion of the participants (76%). This health resource reflects general satisfaction with how the body looks and functions, encompassing physical fitness, physical appearance, and a sense of being pleased with one's body despite ageing.

Physical appearance was highlighted as an integral part of sport participation, particularly in relation to weight control and the possibility of eating and drinking without concern.

I keep my weight despite eating unhealthy things, I sleep better, I feel better. (Man, Orienteering, age 60–64)

Related to weight control and physical fitness, participants also emphasised vanity and looking good despite ageing.

I feel attractive. (Woman, Boule, age 60–64)

I look good – vanity! (Man, Track and field, age 65–70)

Feeling strong and physically fit creates meaningfulness, enabling the participants to feel in tune with their bodies.

In sum, this health resource is about feeling better in your own body, combining physical fitness, physical appearance, and looking good. In contrast to the health resource habit of exercising, the functionality of the body is here related to a general sense of being fit and looking good.

### 3.5 HR5—self-worth

The health resource “self-worth” encompasses self-confidence and comfort with oneself. Approximately one-third of the participants rated this health resource as meaningful in sport participation (32%). Self-worth through involvement in sport also involves autonomy, prioritising oneself and one's health, and feeling confident in one's abilities.

Participants described this health resource as valuing themselves more than earlier in life, when work and family were prioritised. Sport provided confidence and a sense of self-worth.

It gives me the confidence to go out (alone) on dance evenings, e.g., at Gröna Lund, at seniors’ dances, and during the day, at an ‘open house’ in nearby activity centres. (Woman, Dance, no age stated)

Self-worth is also about a feeling of independence and confidence to not give up, and a feeling that you are in control and making your own decisions.

It gives mental stamina; for example, if you win the 3rd set, you get more confidence not to give up in other situations. “I can do it.” If you are strong, you feel safe, you can defend yourself in difficult situations and not be afraid. (Woman, Tennis, age 71–75)

In sum, self-worth as a health resource is about the confidence, autonomy, and prioritising oneself and one’s own health, which are brought about by participation in organised sport.

### 3.6 HR6—identity as an exercising person

The health resource “identity as an exercising person” was rated as meaningful by nearly a quarter of the participants (21%). The health resource reflects identifying as someone who regularly engages in physical activity and serves as a role model for other older adults. Participation in organised sport is thus an important part of life, something to be proud of and to share with friends as an ambassador for exercise.

Participating in physical activity or organised sport was described as a significant part of several participants’ lives, both during retirement and throughout their lives.

It is a large and significant part of my life. (Man, Orienteering, age 71–75)

I have been active in practising, competing as well as being an instructor in judo for 40 years. (Man, Sailing and Budo, age 76–80)

Being a role model for other retired persons was also a big part of the identity as an exercising person. Spreading a positive attitude towards physical activity and organised sport to friends, while communicating that participation in sport and physical activity is a valuable part of life, is an important aspect of identifying as an exercising person.

Being a role model for other older people – to show them that you can continue boat life and sail well into old age (provided you don't get sick, of course). (Woman, Sailing, age 71–75)

The identity and being a role model can also be a feeling of being rational and making sensible decisions in life.

It [doing physical activity] gives me an identity of being a wise person. (Man, Track and field, age 65–70)

And it feels good to be a role model. (Woman, Group exercise training, age 71–75)

In sum, in contrast to self-worth, which is more of an internal health resource, identity as an exercising person is a health resource that is external. It entails identifying as someone who regularly engages in physical activity as well as serving as an ambassador to encourage other older adults to join organised sports.

### 3.7 HR7—capability in and about physical activity

The health resource “capability in and about physical activity” reflects the value of learning, developing skills, improving a technique, or taking on a challenge. This resource was rated as meaningful by approximately one-fifth of the participants (25%). Learning is about the fun of knowing the sport they participate in. This involves performing better and achieving better results than previously, thus developing in the activity.

Developing new skills or improving technical skills was described as both physical and mental, for example skills involved in taking a golf shot and cognitive problem-solving or strategic thinking involved in orienteering. This health resource also includes being challenged to improve physically and mentally, as well as the joy of taking on a movement challenge. For example, learning golf can involve technical skills as well as strategic thinking and problem-solving in navigating a golf course.

The feeling of learning a golf course so you can manage it in fewer strokes or hitting a hole-in-one. (Woman, mini golf, age 65–70)

Developing a skill can also be fun, even if it is not necessarily connected to achieving a particular result.

I am constantly learning to improve my problem-solving skills under physical pressure. (Man, orienteering, age 65–70)

I am getting slightly better with time. (Man, Sailing, age 60–64)

Part of learning and developing skills is about performing better than the previous day, as well as challenging oneself to improve and do better.

I can compare my performance from one occasion to the next. (Man, golf, age 65–70)

It is fun to challenge oneself. (Woman, mini golf, age 76–80)

In sum, capability in and about physical activity is a health resource that includes learning, developing physical and mental skills, performing better, and taking on a challenge.

### 3.8 HR8—competing

The health resource “competing” emerged as the most common of the newly identified resources. Almost half of the comments regarding something other than the originally identified health resources mentioned aspects related to competing. The meaningfulness in this health resource lies in both the results when competing with friends and the value of the competition itself, including the suspense of the moment.

In this health resource, results are essential, serving as a measurement of personal performance, setting new personal bests, or winning. Competing against other older adults, club mates, or peers in competitions was described as important, as was comparing one’s results with that of others.

Competing, being able to compete even though you become older. (Man, Boule, age 65–70)

I have started to compete in veteran classes, which is very motivating! (Woman, Fencing, age 60–64)

Competing against oneself and improving one’s personal best were also important aspects.

That the display on the treadmill makes exercise measurable and comparable from one occasion to the next. (Man, Multisport club, age 76–80)

However, often, the feeling of winning in the moment of competing, as an intrinsic value, is crucial. This is described in terms of the competitive nerve, the contest or the element of competition here and now.

To win! (Man, Boule, age 60–64)

It's fun to compete. It can be added that factors like a better mood and self-confidence in the short term are less relevant on occasions when you lose. (Man, Table tennis, age 60–64)

In sum, competing is a health resource that includes competing against oneself and others, as well as the suspense that competing offers.

### 3.9 HR9—nature experiences

Approximately one-fourth of the supplementary comments described the health resource “nature experiences.” This involves the meaningfulness of nature experiences when participating in sports and physical activities. Such experiences include being out in the fresh air or sunlight, as well as spending time in forests or other beautiful outdoor environments.

In general, this health resource is described as the positive experience of being outdoors and engaging in physical activity.

Being outside and getting fresh air. (Man, Orienteering, age 65–70)

I enjoy being in the forest. (Woman, Orienteering, age 60–64)

Activities specifically related to nature experiences that were mentioned in the survey are walking, orienteering, and golf.

I always feel privileged that I have the opportunity to relax and spend time in the wonderful nature and environment a golf club offers. With or without golf clubs. (Man, Golf, age 71–75)

Orienteering keeps you sharp and is a great exercise also for the mind, and on top of that it is a great way to get out in nature even if I can't run as fast as I did. (Man, Orienteering, age 71–75)

In sum, nature experiences as a health resource conveys the positive connections participants make with nature when participating in sport activities, highlighting positive values associated with being outdoors.

### 3.10 HR10—relationship with animals

Only a few respondents described the health resource “relationship with animals,” but they saw it as the most significant part of why they participated in organised sport activities. This health resource highlights the meaningfulness of the human–animal relationship during physical activity or when participating in organised sport activities. In this resource, the companionship, close contact, and the constant interplay with an animal are essential.

In this study, the relationship with animals primarily involved dogs and horses. Participants emphasised the joy of doing things with an animal and thus forming a special bond. This also involved a love for animals in general.

Everything above [other health resources] is relevant to a higher or lesser degree, but the most important, overshadowing everything else, is the contact with the horse and the riding experience in itself. (Woman, Equestrian sports, age 60–64)

The relationship with the animal was also described as being the ultimate experience or as the extra dimension that made the activity particularly meaningful.

The interplay with the horse, before, during, and after, adds an extra dimension to the exercise. Mentally, when you go into the horse, all other thoughts stop ‘disturbing’ the mind. You have to be present all the time, it is extremely liberating. (Woman, Equestrian sports, age 65–70)

In sum, the health resource relationship with animals involves companionship and interplay with an animal, where the human–animal bond is meaningful in itself, enhancing the physical activity.

## 4 Discussion

In this section, we first discuss the results of our third analytical question, which examined the confirmation and modification of previously identified health resources. We then introduce an empirically based conceptual model, HeRO, to provide an understanding of what health resources older adults find meaningful in their participation in organised sport. Following this, we then discuss our main findings in relation to prior research, before concluding with the strengths and limitations of this study.

### 4.1 Confirmation, modification, and identification of health resources

The original seven health resources were developed in a study with healthy women who participated in a resistance training intervention and continued to meet at the same gym for many years after the intervention ended (11). In that study, the health resources related to physical activity were identified through the ways in which participation contributed to comprehensibility, manageability, and meaningfulness. In the present study, however, a much broader sample was included, with men and women of different ages participating in many different sports. This has provided nuance and extended the previously identified health resources, while also providing the opportunity to identify new ones that older adults find meaningful in organised sport.

In this study, four health resources were confirmed, with only slight nuances identified: social relations (HR2), embodied satisfaction (HR4), self-worth (HR5), and identity as an exercising person (HR6). For example, the health resource identity as an exercising person was strongly emphasised among the women doing resistance training in the earlier study, but in the current study, it was emphasised in some sports such as orienteering, where an orienteerer is something you are, while in tennis, the sport is something you play.

Three health resources were confirmed but extended. The health resource positive energy (HR1) was extended to include enjoyment emphasised in a different way. The women in the earlier study mentioned joy, but only as an extrinsic value. In the present study, joy was often mentioned as an intrinsic value of participating in organised sport. Some participants did not explicitly interpret the health resource positive energy as “just having fun,” so we changed the name of this health resource (HR1) to positive energy and enjoyment. The health resource the habit of exercising (HR3) was extended because of the broader sample. The women in the previous study were physically healthy. In contrast, the broader sample in the present study included some older adults with age-related functional problems. Previously, the health resource emphasised the importance of routines and structure, as well as the role of physical activity in delaying the ageing process. With this new sample, we also identified older adults who perceived routines as important for rehabilitation purposes and for reducing pain. The third health resource extended by the new and broader sample was capability in and about physical activity (HR7). The present study's sample included older adults participating in over 50 different sports, reflecting more dimensions of capability. The health resource was extended to include more aspects of learning and developing in a specific sport, as well as improving techniques and skills to perform better.

Finally, we identified three new health resources: competing (HR8), nature experiences (HR9), and relationship with animals (HR10). These represent distinctly different ways in which the participants conveyed the meaningfulness of organised sport. The health resource competing was not identified in the earlier sample due to the nature of strength training. An obvious explanation here is that the sample consists of sports that you can compete in. The health resources nature experience and relationship with animals were, for obvious reasons, not identified in the previous study. In the present study, sports such as golf, orienteering, and equestrian activities have made this result possible.

### 4.2 Introducing the health resource older adult model (HeRO)

**Figure 1:**
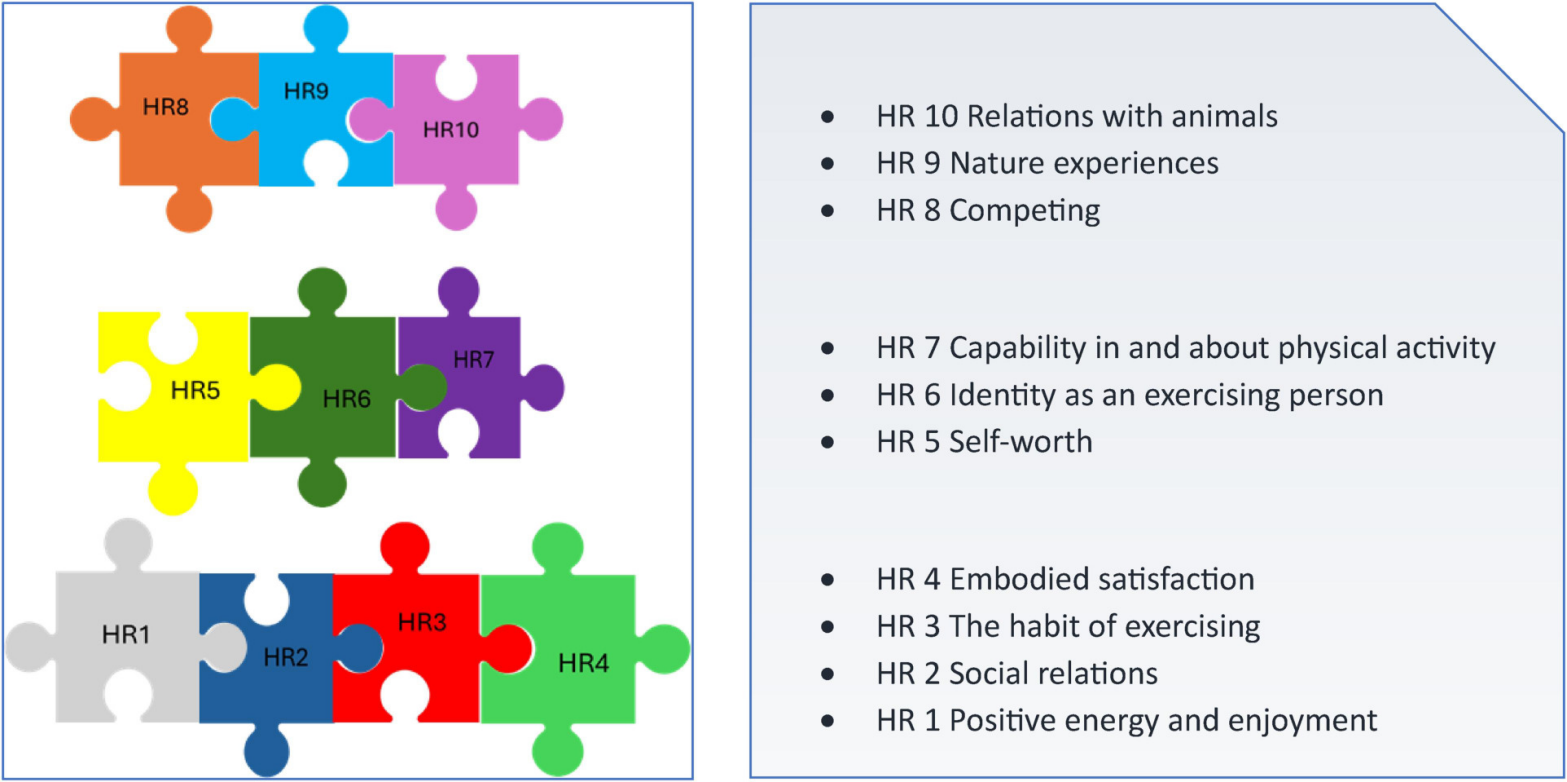
The 10 health resources building the HeRO model are illustrated as 10 different puzzle pieces that can be combined in different ways.

Originally developed from a small group of older women engaged in resistance training (11), then tested in a regional small-scale study on organised physical activity initiatives (18), the model has now been confirmed and extended through a large sample of almost 5,000 older adults. We here introduce HeRO, an empirically based conceptual model (Figure 1) for understanding what health resources older adults find meaningful in their participation in organised sport. The evidence for our claims rests on the following: (i) systematic methods tested in previous studies; (ii) a large, purposeful sample balancing generalisability and uniqueness; (iii) a theoretically grounded analysis; and (iv) evaluation against Tracy's (20) end goals for excellent qualitative research. In this manner, we argue that we have provided strong empirical support for the claims that underpin the proposed model.

The HeRO model can be understood as 10 different, but equally sized, puzzle pieces that can be combined into individually shaped puzzles. Each puzzle reveals which health resources older adults find meaningful in their participation in organised sport. Most puzzles, but not all, are based on the four most common health resources: positive energy and enjoyment (HR1), social relations (HR2), the habit of exercising (HR3), and embodied satisfaction (HR4). In other words, it is common for older adults to highlight having fun, socialising with others, practising sport as a routine, and feeling fit as health resources gained from sport.

**Figure 2:**
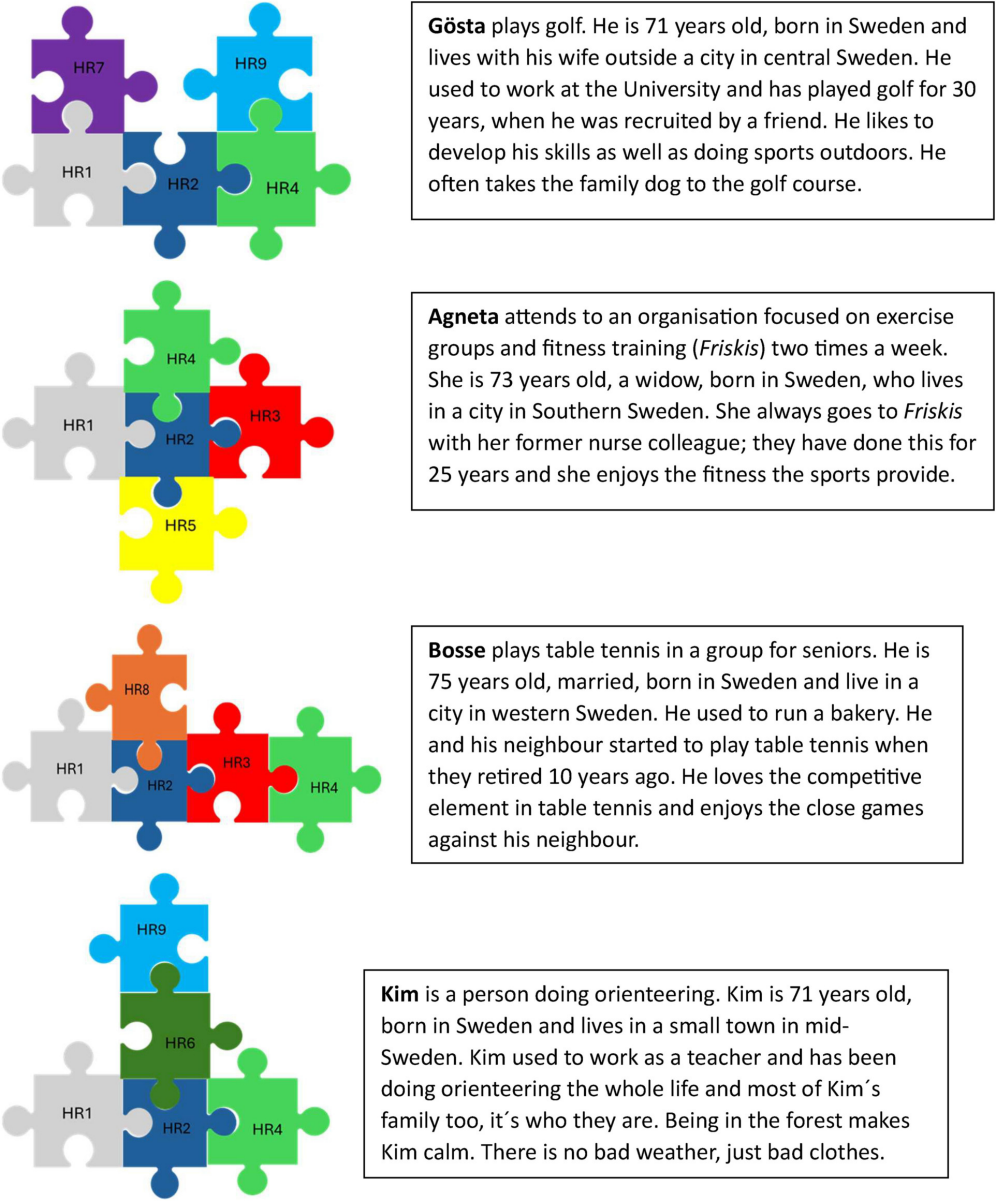
Four fictional older adults illustrated as different HeROs. The synthesised examples are derived from the empirical material and visualise the differences in what people find meaningful in sport.

Different people also convey some of these four health resources in combination with other resources, such as: feeling of confidence (HR5), because it is who they are (HR6), to develop new skills (HR7), to win today's match (HR8), to get fresh air (HR9), or to spend time with an animal close to your heart (HR10). Hence, each older adult has a different puzzle that, through a combination of health resources, explains their participation in sport. As an illustration, we introduce four fictional older adults drawn from our empirical material: Gösta, Agneta, Bosse, and Kim (Figure 2). The added value of the model, as shown in the synthesised examples, is that it depicts everyone as different, with their own unique combination of puzzle pieces. For example, some people may not have any of the most common HR1–HR4 pieces in their puzzle.

### 4.3 What does this model add?

As stated in our background, results regarding older adults’ participation in sport depend on (i) what constitutes organised sport; (ii) who is considered an older adult; and (iii) what questions the studies ask. While previous studies have mostly focused on physical health outcomes of physical activity (e.g., 3, 4, 5, 6), and sometimes on older adults who never take part in sport (2, 15), our model is based on a large and diverse sample of older adults who participate in many different sports.

Most importantly, the third issue—namely, that the results of studies often depend on what researchers ask, particularly in relation to health—is where our model contributes the most. The HeRO model is based on a salutogenic approach of asking questions about what health resources older adults find meaningful for participation in organised sport. In this study, we confirmed, modified, and identified health resources using salutogenic theory (13). In this manner, we moved beyond understanding the relationship between participation in sport and health as merely being about levels of physical activity. In many studies on health and sport, the underlying assumption is that health is an outcome of sport because it involves physical activity (16). Instead, by asking salutogenic questions, health in our model is understood as a broad concept, including physical, mental, and social aspects. This conveys both preventive and promotive motives, as both a means and an outcome, and also being able to entail both individual reasons and the individual in relation to the organisational level.

For example, in our model, competing (HR8), as also highlighted by Heo et al. (10) and Stenner et al. (5), is a health resource since it makes participation in sport meaningful for some older adults. Another example is the health resource the habit of exercising (HR3). Here, the habit includes sport as a means to prevent risks of ill-health but also the intrinsic quality in daily life here and now from participating in sport. Using salutogenic theory also makes it possible to understand that participation in sport goes beyond individual motives or outcomes. Instead, participation is always in relation to other people and the activity itself, highlighting, for example, competitive elements or contextual elements such as nature or animals.

There are, of course, other models and reviews in the literature that explain older adults’ participation in sport and physical activity. Some are more delineated, focusing on specific target groups or sports (5, 9), and others, often based on literature reviews, are quite comprehensive (8, 21). Comparing three reviews (5, 8, 21) with our empirically based conceptual model, we find that many of what we call health resources are factors or reasons also mentioned in these reviews. For example, Morgan et al. (21) and Meredith et al. (8), who also focus on broad groups of older adults, include most of our health resources, but not all. Morgan et al. (21) conclude that an overly focused emphasis on the protective health benefits of physical activity can be counterproductive to encouraging older adults to engage in it. In the review, an assumed linear relationship regarding physical activity as a means to health benefits hampers older adults’ participation, as this may not provide sufficient motivation for them to engage. With a broader understanding of what constitutes health benefits from participation, more older adults may be encouraged to engage in and reap the benefits of physical activity. Stenner et al. (5), in their review, explore reasons for older adults’ participation in sport, but seem to assume that health-related benefits are the primary motivation for continued participation. Meredith et al. (8), on the other hand, in their review, examine factors influencing physical activity engagement and distinguish between physical activity as pleasurable movement and as a health-related anti-ageing behaviour.

However, viewing sport as primarily a means to physical health benefits risks an emphasis on arguments related to ill-health or age-related declines in physical abilities (5). Instead, what we add by asking about what is meaningful in their participation and engagement in sport, is that different health resources can be identified from more general resources to more sport or activity-specific resources. Hence, the added value of the HeRO model is that it provides a flexible perspective, depicting all older adults as diverse individuals.

### 4.4 Strengths and limitations

The use of a large sample of both men and women, from all over Sweden, participating in many different sports, has provided a unique opportunity to build the HeRO model. Another strength is the theory-based approach, which builds on earlier work by our research group. However, a limitation of this study is the homogeneous sample of older adults. Over half of the participants had higher education, and very few people were born outside Sweden. This is not a representative sample of the Swedish older population, even though it appears to be representative of older adults participating in sport in general (22). This may constrain the generalisability of these findings to the wider population of older adults, particularly those who may no longer be engaged in sporting activities. However, we can claim to have a reasonable sample of older adults who participate in organised sport in Sweden. At the same time, Sweden, like many other countries, faces the challenge of making sport participants more heterogeneous. A follow-up study using this material will therefore focus more on the differences between health resources, individuals, and what sport they participate in.

Another important aspect is the definition of sport and, consequently, who is included in studies on participation in organised sport. Here, we succeed in some ways but also fail in others. In Sweden, organised sport is most often administered under the Swedish Sports Confederation, although with a broad definition of what sport is. However, for this particular target group, organised physical activity initiatives (which are sometimes very similar to sports and hard to separate) can also be organised by senior associations and ethnic organisations. These were not part of the sample in this study, but have been included in earlier studies on which this research builds (23).

A further limitation is that not all 10 health resources were tested in the same way in the large sample, since only seven were included in the original questionnaire (SPAHRQ), making it difficult to ascertain, for example, how common these resources are or to what extent competing is considered meaningful. At the same time, by using open-ended questions, we were able to identify new health resources from comments from a smaller sample. We suggest that future studies use all 10 health resources in an extended SPAHRQ to better capture the full picture of sport participation among older adults. Using this model in other countries and in more heterogeneous groups could also add new dimensions to our framework.

## 5 Conclusion

This study concludes that older adults’ participation in organised sport can be explored and understood as a combination of what they consider meaningful in their engagement. Participation involves a combination of 10 health resources, where the majority of older adults highlight having fun, socialising with others, maintaining sport as a routine, and feeling fit as their main reasons for engaging in sport.

We contend that what older adults perceive as meaningful should guide how sport is organised, enabling more older adults to reap the benefits of participation. This also has important implications for public health interventions and primary care for older adults in terms of how to motivate older adults to become physically active in sport beyond arguments for increased activity levels. Moreover, what older adults think is meaningful is not the sole answer to how sport should be organised. However, since the health resources in this study have been developed from a broad concept of health—taking into account both the individual and the environment, and the relation between the two—the HeRO model covers more aspects than other models. Importantly, different older adults prefer different things in order for sport to be meaningful. The HeRO model can thus be helpful as a flexible, empirically based conceptual framework that takes more pieces of a complex puzzle into account.

## Data Availability

The data supporting the conclusions of this article will be shared on reasonable request by the corresponding author.
